# Per Aspera ad Chaos: Vladimir Uversky’s Odyssey through the Strange World of Intrinsically Disordered Proteins

**DOI:** 10.3390/biom13061015

**Published:** 2023-06-19

**Authors:** Prakash Kulkarni, Stefania Brocca, A. Keith Dunker, Sonia Longhi

**Affiliations:** 1 Department of Medical Oncology, City of Hope National Medical Center, Duarte, CA 91010, USA; 2 Department of Systems Biology, City of Hope National Medical Center, Duarte, CA 91010, USA; 3 Department of Biotechnology and Biosciences, University of Milan-Bicocca, 20126 Milan, Italy; 4 Biochemistry & Molecular Biology, Indiana University School of Medicine, Indianapolis, IN 46202, USA; 5 Architecture and Function of Biological Macromolecules (AFMB), UMR 7257, Aix Marseille University and CNRS, 13288 Marseille, France

## 1. Introduction

Until the late 1990s, we believed that protein function required a unique, well-defined 3D structure encrypted in the amino acid sequence. However, over the past two decades, we have witnessed a protein ‘renaissance’. We learned that proteins with unique 3D structures can switch folds (referred to as ‘metamorphic’ or shapeshifting proteins), and a new class of proteins, called intrinsically disordered proteins (IDPs), that lack structure but can either transition to order, or in many cases, be functional in the absence of any structure (‘fuzzy’ logic), was discovered. Thus, contrary to the hitherto prevailing dogma, it became apparent that protein structure cannot be envisaged merely as binary states; rather it is a continuum of conformations including the propensity to form amyloid fibrils and encode information for transgenerational inheritance. Furthermore, in the last decade we realized that many IDPs and prion-like proteins have the potential to undergo liquid-liquid phase separation (LLPS), a process underlying the formation of so-called proteinaceous membrane-less organelles (PMLOs). Because PMLOs serve as master regulators of the cell, the propensity of IDPs to undergo LLPS means that a role for IDPs in evolution of life on earth can be argued, especially prebiotic evolution.

Thus, it follows that a new era in the protein field has dawned in which IDPs have attracted a lot of interest. Nonetheless, IDPs also pose a major challenge as they are not easily tractable experimentally using techniques and approaches traditionally used by protein scientists. Because of this, IDPs, which are prevalent in all three domains of life and comprise almost half the human proteome, are often referred to as constituents of ‘dark matter’ in biology.

## 2. A Special Issue in Honour of Vladimir Uversky

This Special Issue of Biomolecules, “Physics of Protein Folding, Misfolding, and Intrinsic Disorder: A Themed Issue in Honour of Professor Vladimir Uversky on the Occasion of His 60th Birthday”, is a dedication to one of the discoverers of IDPs [1]. This collection is a small token of the respect, admiration, and affection the contributors have for Prof. Vladimir (Volodya) Uversky. It is also a celebration of his illustrious career, and his accomplishments, and contributions to the IDP field that have inspired so many minds worldwide.

This Special Issue presents the state of the art as it emerges from the contribution of the community of IDP researchers (IDPers), who have responded to the invitation to give credit to the pioneering work of Prof. Vladimir Uversky aimed at defining the class of disordered proteins and at promoting the attention of scientists toward the existence of “non-globular proteins”.

**Figure 1:**
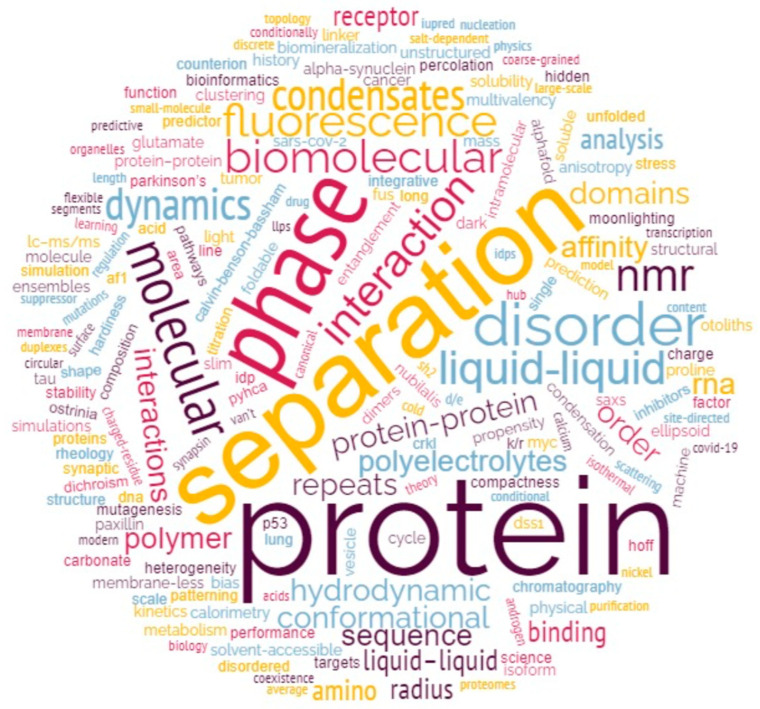
Cloud analysis of key words of papers of the Special Issue in honour of Prof. Vladimir Uversky.

The papers in this collection show the advancement of our knowledge through the application of an integrative structural approach and witnesses at the same time the interest of the IDPer community toward new concepts (i.e., liquid-liquid phase separation) and new methodological frontiers (i.e., the application of machine learning and artificial intelligence to disorder prediction and modelling). This Special Issue collects 20 original research articles and 5 reviews, divided into 4 main areas (see below), which we defined for the purpose of this editorial by drawing on cloud analysis applied to the whole list of keywords (Figure 1).

## 3. The Different Areas and the Distribution of Articles within Them

*Intrinsic disorder characterization and methodological development*—Integrative structural biology, which is the application of multiple experimental and computational methods, has emerged as an essential approach to understanding IDP phenomena. The collection of papers and reviews in this section embodies this philosophy to study IDPs. This appears as one of the most intense fields of study, with 6 scientific articles and one review [2,3,4,5,6,7,8] (Table 1).

*Phase separation*—Liquid-liquid phase separation (LLPS) represents one of the major functional areas in which IDPs are involved and has been attracting a lot of interest in the scientific community in recent years. This is also evident from the numbers of contributions on this topic received in our SI, with five scientific articles and three reviews [9,10,11,12,13,14,15,16] regarding both computational prediction and biochemical analysis of condensation propensity.

*Binding mode and properties of IDPs/IDRs*—IDPs are remarkably well suited to interact with other proteins and are often found at the center of interaction hubs. Understanding the subtle mechanisms by which such interactions are triggered and regulated is of paramount importance to decipher their pathophysiological role, but also for exploring the potential for pharmacological approaches targeting IDPs. Under this theme, readers will find five scientific articles and one review [17,18,19,20,21,22].

*Predicting and modeling of IDPs by conventional and advanced bioinformatic tools*—Significant progress has been made over the past decade in the development of bioinformatics tools for predicting and modeling structural disorder. In addition to conventional predictors, which often make use of compositional bias analysis, one increasingly finds artificial intelligence-based programs that enable accurate and rapid analysis of entire proteomes. Four articles can be found in this section of SI [23,24,25,26]. 

In that respect, note that half of the papers focused on phase separation address the phenomenon with computational and bioinformatic tools.

## 4. The Impact of Vladimir Uversky on the Scientific Community and Our Careers

The full range of Volodya’s contributions is so vast to describe that it would be a disservice to him if we even tried to do so in this editorial. Nonetheless, despite the reputation he has earned, Volodya is one of the most humble, respectful, and caring scientists you will come across. A few statistics concerning Volodya and the profound influence he has had on the field is worth mentioning to justify how flabbergasted one may feel when meeting him for the first time; Volodya has published ~1150 research articles and reviews, ~110 book chapters, edited or co-edited >25 books, edited 5 book series that include 45 volumes, guest edited countless Special Issues for various Journals, and mentored/advised ~185 undergraduate, masters, graduate students, postdoctoral fellows and visiting faculty over his career spanning over 3 decades. As of 2021, Volodya had co-authored papers with >15,000 researchers from >2750 institutions in 90 countries around the globe!

As Guest Editors of this Special Issue, we are indeed delighted to honor an esteemed colleague and friend, but to illustrate his humility, dedication, and gregarious personality, we have taken the liberty to share a few personal anecdotal notes with the readers.

Prakash Kulkarni: I have known Volodya for almost a decade and we have published several papers together. However, if I were to highlight 1 or 2 papers that I would consider as the most significant and thought provoking among them, the one elaborating the concept of IDPs as complex systems, would be one. Beginning 2010, together with Govindan Rangarajan, my mathematics collaborator, we were working on the IDP conformational noise hypothesis. At the time, I was toying with the idea that IDPs that are critical in events such as phenotypic switching and cell fate determination, may be viewed as dynamical systems. Our conformational noise paper [27] was published on 19 December 2012, and thus, we figured we may resume working in January 2013 right after the Christmas holidays. However, we were surprised when, on 23 December 2012, we saw Volodya’s paper also alluding to IDPs as edge-of-chaos systems [28]! In retrospect, we should have anticipated this knowing very well that Volodya is a physicist!

At any rate, I decided that we should reach out to him and perhaps, eventually join forces to explore these initial ideas further. And so, we did. And true to his impeccable reputation, he not only agreed to hear me out, but also shared his insight and some ideas on how we may want to approach the problem. The paper we published in Chem Rev together with Rangarajan and several of our other colleagues was the culmination of this enduring spirit of cooperation [29]. A couple of other papers that we published together which I cherish immensely are the role of IDPs in evolution using the beak of the finch and the origin of multicellularity in the green algae, as paradigms [30,31]. The thrill and the excitement we shared when we conceived all these ideas prior to putting them down as formal manuscripts is hard to describe in words. Nonetheless, I have not had an opportunity to meet Volodya in person thus far and I look forward to that day in earnest.

Stefania Brocca: I first came into contact with Volodya in 2008, when the existence of IDPs already seemed to be fairly accepted by the international scientific community, but still none of the researchers in my department had had the opportunity to come across them. The opportunity to collaborate with Volodya jumped out through a study on Sic1, a yeast cell cycle regulator, as part of a project of which Prof. Lilia Alberghina was PI. So, we started a biochemical characterization project on a protein that was “strange” compared to those hitherto manipulated by myself and colleagues in my department. The first question to be answered concerned the prediction of disorder. I first turned to Dr. Sonia Longhi, a friend and former lab colleague in Prof. Marina Lotti’s group, who did not hesitate to suggest contacting Volodya. I had little confidence that a “super-star” scientist of his stature could devote time to an obscure project of an unknown Italian researcher. Encouraged by Sonia, I wrote to him, and Volodya, to my surprise, not only responded immediately and effectively, but treated me with unparalleled helpfulness and friendliness. Volodya was a co-author of that paper [32], which was the first of a series of papers [33,34,35,36,37]. These collaborative efforts also involved other colleagues in my Department, namely Prof. Rita Grandori, Prof. Silvia Maria Doglia, Dr. Antonino Natalello, and Dr. Carlo Santambrogio, experts in biophysical techniques such as native mass spectrometry and Fourier transform infrared spectroscopy. The collaboration with Volodya was instrumental in the creation and consolidation of our multidisciplinary team for the study of IDPs. Alongside IDPs, Volodya has also been involved in studies on protein folding and aggregation, starting to be recognized as an issue of biological and biotechnological interest at that time [38,39]. I have met Volodya personally only a couple of times. Nevertheless, his energy, enthusiasm and availability make him an ever and effectively present member of my Dept’s team of IDPers.

Alan Keith Dunker: In early 1999 the *Proceedings of the National Academy of Sciences*, USA (PNAS) asked me to review a manuscript submitted by Volodya and co-workers. This paper showed that structured proteins and IDPs could be separated by a straight line on a plot of a protein’s net charge (*Y*-axis) versus its overall hydropathy (*X*-axis) using the Kyte and Doolittle hydropathy scale. On this “Charge-Hydropathy Plot”, IDPs are distinguished from structured proteins by their higher net charge and reduced hydrophobicity.

By early 1999 we had already published our early predictors of protein disorder and we were working on improving them. Our published and unpublished data supported the overall findings in Volodya’s PNAS submittal, but Volodya had missed our papers. After pointing out these missing references, we gave Volodya’s submittal a very strong positive review. To my great surprise, this paper was not accepted by PNAS. Several years later one of the world’s leading biochemists told me that he rejected Volodya’s PNAS submittal and that he regarded this as one of his biggest mistakes. Indeed, that preeminent biochemist has published multiple papers on IDPs!

Volodya’s PNAS submittal was eventually published in late 2000 in the journal Proteins [40]. In this version of his paper, Volodya not only included the missing references to our work, but he also showed that, for a particular set of IDPs, his Charge-Hydropathy Plot and our predictions agreed with each other.

Sometime after “meeting” Volodya via his PNAS paper submission, I contacted him and arranged to meet him in person at the Annual Meeting of the Biophysical Society in San Francisco on 23 February 2002. We expressed our interest in working together.

In July 2003, I moved from Washington State University to Indiana University School of Medicine to become the founding Director of the Center for Computational Biology and Bioinformatics. Recruiting Volodya became a very high priority. He joined me in Indiana in 2004 and remained with me there until 2010. During this period and continuing to the present, we have published more than 100 papers together. I have never met anyone who works even half as hard as Volodya does.

At Volodya’s suggestion, we revisited his Charge-Hydropathy Plot to test whether alternative hydropathy scales might improve the predictions. We compared the Kyte-Doolittle scale to 18 other published scales, the best of which proved to be a scale developed by Robert Guy. In addition, we developed a new scale called IDP-Hydropathy. The balanced accuracies of the Charge-Hydropathy Plot using each of these scales are 79 ± 9% (Kyte-Doolittle), 84 ± 9% (Guy), and 90 ± 7% (IDP-Hydropathy), where balanced accuracy is given by (%Correct Structure + %Correct Disorder)/2 [41].

Even though Volodya left Indiana in 2010, we continue to collaborate [42,43]. Words cannot express how much Volodya has helped me and our students over our many years together.

Sonia Longhi: My first contacts with Volodya date back to 23 years ago, when my PhD student at that time contacted Volodya to clarify a doubt, we had on how to compute the actual hydrodynamic radius versus the one expected for an IDP from the gel filtration profile. We were very impressed by the rapidity of his answer and by his accessibility and patience. Thanks to his clarification and explanation, we could bring the last finishing touches to the study we were carrying out [44]. Shortly thereafter, we benefited from his deep knowledge of IDPs and co-authored a review on the assessment of protein disorder and induced folding that is still nowadays highly cited [45]. This was the first of a total of 13 co-publications over years, including a comprehensive review on intrinsic disorder [46] and three publications that also involved another guest editor of this Special Issue (i.e., Prof. Stefania Brocca) [34,36,37]. On top of that, we co-edited a book on experimental methods for IDPs [47] and a book on structural disorder within viruses [48]. In the context of all these collaborations over the years I have been dumbfounded by his working abilities and reactivity. His responses were so prompt that I had the impression he was working in the neighboring office, and we were actually chatting. This has now become a sort of joke between us, whereby in the last exchange, no more than a few days ago, I noticed that he was “getting slower” (his answer took three minutes instead of the usual 30 s!), a “slowness” that Volodya ascribed to the fact that he had just turned 60!

Our first encounter dates back to 2007, on the occasion of the first meeting of the IDPs subgroup of the Biophysical Society in Baltimore, a subgroup which was created at the initiative of Volodya and Prof. Keith Dunker. Since then, I have had multiple occasions to meet him, including when he accepted my invitation to visit my lab and give a highly appreciated seminar in 2011. Over the years, he proved to be always extremely supportive and eager to help. His work has been very inspiring to me. Having started myself working in the field of IDPs in the early 2000s, I can perfectly appreciate the difficulties that he must have encountered to make the concept of disorder accepted in the scientific community. I am very admirative of his perseverance and of all the efforts he did to make this concept be adopted by protein scientists. Without his efforts and energies, I doubt I would have dared to make the decision of becoming an “IDPer”. Thanks, Volodya, for having been such a pioneer and for your guiding role!

We wish Volodya a very happy 60th birthday, and a long healthy life in the years to come, and keenly look forward to seeing more discoveries by him.

## Figures and Tables

**Table 1:** Content of the Special Issue in honour of Prof. Vladimir Uversky. Four main topics can be identified into which the 25 articles are distributed.

Intrinsic Disorder Characterization & Methodological Development	
A Novel Tandem-Tag Purification Strategy for Challenging Disordered Proteins	Mészáros et al., 2022 [2]
Illuminating Intrinsically Disordered Proteins with Integrative Structural Biology	Evans et al., 2023 [3]
Distribution of Charged Residues Affects the Average Size and Shape of Intrinsically Disordered Proteins	Bianchi et al., 2022 [4]
Identification of Intrinsically Disordered Proteins and Regions in a Non-Model Insect Species *Ostrinia nubilalis* (Hbn.)	Avramov et al., 2022 [5]
NMR Reveals Specific Tracts within the Intrinsically Disordered Regions of the SARS-CoV-2 Nucleocapsid Protein Involved in RNA Encountering	Pontoriero et al., 2022 [6]
The Ni(II)-Binding Activity of the Intrinsically Disordered Region of Human NDRG1, a Protein Involved in Cancer Development	Beniamino et al., 2022 [7]
Deciphering the Alphabet of Disorder—Glu and Asp Act Differently on Local but Not Global Properties	Roesgaard et al., 2022 [8]
**Phase Separation**	
An Interpretable Machine-Learning Algorithm to Predict Disordered Protein Phase Separation Based on Biophysical Interactions	Cai et al., 2022 [9]
Quantifying Coexistence Concentrations in Multi-Component Phase-Separating Systems Using Analytical HPLC	Bremer et al., 2022 [10]
In-Silico Analysis of pH-Dependent Liquid-Liquid Phase Separation in Intrinsically Disordered Proteins	Pintado-Grima et al., 2022 [11]
Different Forms of Disorder in NMDA-Sensitive Glutamate Receptor Cytoplasmic Domains Are Associated with Differences in Condensate Formation	Basak et al., 2022 [12]
Effects of Mass Change on Liquid–Liquid Phase Separation of the RNA-Binding Protein Fused in Sarcoma	Dong et al., 2023 [13]
Topological Considerations in Biomolecular Condensation	Das and Deniz, 2023 [14]
Reorganization of Cell Compartmentalization Induced by Stress	Fefilova et al., 2022 [15]
The Role of Intrinsically Disordered Proteins in Liquid–Liquid Phase Separation during Calcium Carbonate Biomineralization	Tarczewska et al., 2022 [16]
**Binding Mode and Properties of IDPs/IDRs**	
Portability of a Small-Molecule Binding Site between Disordered Proteins	Jaiprashad et al., 2022 [17]
The Role of Membrane Affinity and Binding Modes in Alpha-Synuclein Regulation of Vesicle Release and Trafficking	Das et al., 2022 [18]
Sequence Properties of an Intramolecular Interaction that Inhibits p53 DNA Binding	Gregory & Daughdrill, 2022 [19]
Folding and Binding Mechanisms of the SH2 Domain from Crkl	Nardella et al., 2022 [20]
Linker Length Drives Heterogeneity of Multivalent Complexes of Hub Protein LC8 and Transcription Factor ASCIZ	Walker et al., 2023 [21]
A Trajectory of Discovery: Metabolic Regulation by the Conditionally Disordered Chloroplast Protein, CP12	Gérard et al., 2022 [22]
**Modeling of IDPs by Conventional and Advanced Bioinformatic Tools**	
Digging into the 3D Structure Predictions of AlphaFold2 with Low Confidence: Disorder and Beyond	Bruley et al., 2022 [23]
The Difference in Structural States between Canonical Proteins and Their Isoforms Established by Proteome-Wide Bioinformatics Analysis	Osmanli et al., 2022 [24]
Conformational Analysis of Charged Homo-Polypeptides	Bigman and Levy, 2023 [25]
Compositional Bias of Intrinsically Disordered Proteins and Regions and Their Predictions	Zhao and Kurgan, 2022 [26]
